# Correspondence

**Published:** 1901-08

**Authors:** N. T. Howard

**Affiliations:** Dahlonega, Ga.


DAHLONEGA, GA., July 11, 1901.

*Editor Atlanta Journal-Record of Medicine:*

I write you a note to ask a personal explanation.

In the January number, 1901, of THE ATLANTA JOURNAL-RECORD OF MEDICINE, pages 663, is published an article of mine upon the merits of Tinct. of Gum Guaiac in the treatment of disease. In this article several times is mentioned *ammoniated* tincture of Gum Guaiac, when I intended the use of the term "Dewee's Compound Tincture of Gum Guaiac or Allied Tincture. By mistake or want of sight on my part, ammoniated tincture was not stricken from the draft of the article.

Will you give this correction a place in your publication, and oblige,

N. T. HOWARD, M.D.,
Dahlonega, Ga.

